# Sleep as a Poison

**Published:** 1870-02

**Authors:** 


Sleep as a Poison.—There are slow and silent influences operating upon our lives, whose activity, though we are constantly subject to, we little estimate. One-third of our existence is under their control, and the remainder depends, in a great measure, for its health, upon the proper use of and benefit we derive from that neglected portion. Insurance agents may safely knock off one-half the premium on that man's policy who has learned to sleep well—that rises, morning after morning, thoroughly refreshed in body and mind; with whom the sun of an unclouded system springs forth to the duty of the day; whose limbs, and voice, and look, all indicate recuperation and preparation.

Sometimes the rich resources of nature may permit over drafts, but even then we tax the future, while we rest in the enjoyment of the present, for in the day-book of life the constantly accumulating little charges inevitably find their way to the ledger, and, at some time, require a balance at our hands, when least we are prepared for the emergency.

Sleep not only has to make up for the wasted powers, but supply the material for their further use; and in proportion to our remembrance of this latter object, will be our prospect of health and continued life. The pressing calls of fatigue and hunger we can not overlook—they are too urgent to be neglected—but a vitiated atmosphere, sapping continually the power of recuperation, and tending to disorganization of all the component parts of the system, receives little practical attention.

The room that we prepare for our use during the time that the being is least able to defend itself from the attacks of disease, is too often the smallest and most unhealthy in the house. Want of space to breathe makes our sleep synonomous with stagnation.

The laws of ventilation can never be properly subjected to use excepting in large apartments, as in smaller rooms we find that the necessary change sufficient to procure renewal of air, produces draught, a state of exposure necessary to be avoided.

If we were desirous of shortening the period of our existence, of paving the way for every affliction that flesh is heir to, of reducing the means of resistance to the inroads of every human malady, we would select the least room in our tenement, and gather together there the greatest number of air consumers. We should expect to come forth from our temporary coffin with blanched hue, and feeble step, with digestion impaired, and blood loaded with impurity. If, under such provocation, we can not reduce our refractory system to perform the daily process of digestion, assimilation, circulation and secretion, by stimulating medicines, we can still further debilitate our flagging powers. At last, when old age or disease comes on us apace, though our years are not long in the land in one sense, in another, we find no lion constitution to cover our defects. View the benefit to be derived from a proper and judicious attention to the restorative power gained by healthy sleep, and we can perceive its true value.

Sleep is the suspension of sensation and voluntary motion necessary to the recuperation of the nervous system, and the due supply of nervous power for continued action. During the repose that brings suspension of exhaustion and waste, by the purity of the air we breathe not only is the circulation kept in its integrity, but the due nutrition of the system, by means of the involuntary organic functions, is also carried on in a state of activity.

One good night's sleep has not only restored the flagging energies, but often has been the critical juncture that has saved life. Rest for the tired limbs does not bring the marked change in disease that comes with abundance of fresh air, as has so often been shown to us. The accession of vitality, by the plentiful supply of fresh air, in thousands of instances during the late war, has verified the remark of a general, that "under a tree he always found the best hospital." A man's wealth is of but little value to him if he can not afford the positive luxury of pure air. That, of all others, should be the central thought of home architecture, and all such influences as would tend to hinder the free winds of Heaven, or load them with poisonous exhalations, should be banished from the apartment where nature has the only time, alone and untrammeled, to correct the mistakes of our active life. Space for breath, without as well as within, and the sickly, nervous, consumptive dispeptic, the bilious, morbid gloom of poisoned circulation, and the feeble muscular inactivity, will give place to elasticity and hope.

In this country, especially, is a fire-place necessary for the proper ventilation of the sleeping apartment, and the most perfect one that can be devised, while in sickness it is always ready for use. Stoves test the endurance of the human lungs as surely as they contaminate the air, and that form is yet to be invented that will combine safety, comfort and ventilation. The open fire-place, with its ascending and descending currents, if it be properly constructed, preserves the necessary equilibrium of warmth, freshness and cheerfulness, that should make the sleeping apartment the pleasantest in the house.—*Oregon Medical and Surgical Reporter.*

